# Impact of Food Intake and Sleep Disturbances on Gut Microbiota

**DOI:** 10.7759/cureus.70846

**Published:** 2024-10-04

**Authors:** Vignesh S D, Vijayakumar T M, N Sai Supra Siddhu

**Affiliations:** 1 Department of Pharmacy Practice, Sri Ramasamy Memorial (SRM) College of Pharmacy, SRM Institute of Science and Technology, Kanchipuram, IND

**Keywords:** diet, food, gut microbiota, probiotics, sleep disturbances

## Abstract

*Lactobacilli* and *Bifidobacteria *are types of microbiota that live in the gastrointestinal (GI) system, or gut microbiota, and are essential for both human well-being and disease. This review looks at the relationship between the composition of gut microbiota and function and two important lifestyle factors - dietary intake and sleep disorders. The diversity of the gut microbiota and metabolic processes is strongly influenced by food intake. Fiber-rich diets encourage the development of good bacteria that synthesize short-chain fatty acids (SCFA), while diets heavy in fat or sugar can negatively impact the microbial balance. Microbial communities are also impacted by regular meal schedules and probiotic and prebiotic use. Sleep disturbances cause stress reactions that alter gut microbiota and upset circadian rhythms. These include irregular sleep cycles and insomnia. These effects are driven by the immune system and gut-brain axis dysregulation, which affects microbial diversity and plays a role in GI and metabolic illnesses. The significance of comprehensive lifestyle treatments to improve gut health is highlighted by an understanding of these interconnections. It may be possible to modify the composition of gut microbiota and improve general health outcomes through the use of strategies that emphasize balanced meals, consistent eating schedules, and better sleep hygiene.

## Introduction and background

The health of our bodies relies on the condition and function of the digestive or gastrointestinal (GI) tract. After the lungs, it has the second-largest area of coverage in the body, covering an estimated 250-400 m^2^, approximately the size of a tennis court. Throughout an average lifetime, about 60 tonnes of food and various environmental microbes pose a significant threat to the integrity of the human GI tract [1]. Over 10^14^ microbial cells consisting of around 1,000 different species of bacteria are present in the human GI system, predominantly in the colon. The gut microbiota, a collection of eukarya, archaea, and bacteria that live in the GI tract, has evolved through millions of centuries of co-evolution into a complicated and mutually beneficial connection with the host [2]. The gut microbiota is essential to the growth of the host's protection, food digestion, gut endocrine function regulation, nervous system signaling, drug effect and metabolism modification, toxin elimination, and the synthesis of numerous substances that affect the host. The four phyla *Firmicutes*, *Bacteroidetes*, *Actinobacteria*, and *Proteobacteria* make up the majority of the human gut microbiota. Rich in microbial DNA, with a stable core of bacteria, a healthy microbiota has a high taxonomic diversity. The quantity of microbes in our gut microbiome is about equivalent to the quantity of somatic cells in our bodies, and these microbes have discernible effects on the majority of metabolic processes that occur in vivo [3]. The metabolism of nutrients, especially the fermentation of complex carbohydrates and dietary fibers that are difficult for the host enzymes to break down, is one of its primary functions. Short-chain fatty acids (SCFAs) are critical for maintaining the integrity of the intestinal barrier and controlling the immunological response as well as for colonic epithelial cells' energy supply. The three main SCFAs produced are acetate, propionate, and butyrate. For human colonocytes, butyrate serves as their main energy supply. Additionally, it can trigger intestinal glucose production, which is beneficial for preserving glucose and energy homeostasis, and cause colon cancer cells to undergo apoptosis. [4]. These microbes perform a number of vital functions, such as providing vitamins and other nutrients, fighting off infections, and supporting the preservation of the mucous membrane. Above all, the gut microbiota communicates with the immune system, delivering cues to support immune cell maturation and the appropriate progression of immunological functions [5].

**Figure 1:**
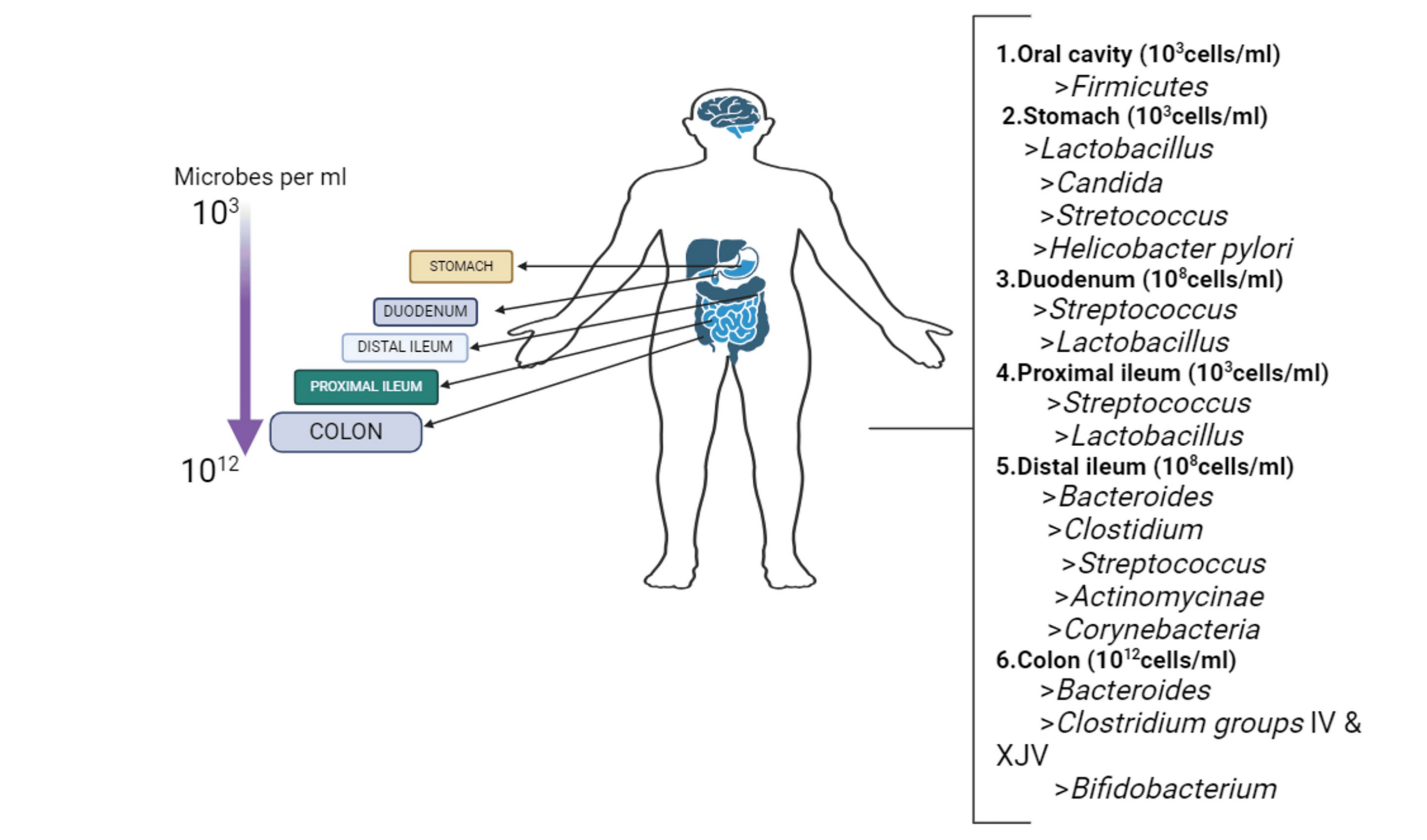
The composition of microbiomes at various body locations in humans. Image created using BioRender (https://biorender.com/).

The three categories of gut microbiota are pathogenic bacteria, conditional pathogenic bacteria, and beneficial microorganisms. The predominant bacteria in the digestive system, known as beneficial bacteria, are involved in immune control and intestinal homeostasis maintenance. An imbalance in the beneficial bacteria arises from an overgrowth of harmful bacteria, leading to disease [6]. Numerous illnesses linked to the gut microbiota include metabolic syndromes, diabetes, autism, inflammatory bowel disease, intestinal infection, inflammatory bowel disease, liver disease, GI cancers, and functional GI disorders. These develop when homeostasis is compromised by quantitative or qualitative changes in the gut microbiota [7]. The gut microbiota that is found in the intestines and is impacted by several factors, including the host's genetic makeup, habits, and mode of birth has a profound effect on humans. Owing to several elements such as nutrition, pH levels, intestinal motility, and host secretions like mucus, bile, gastric acid, and digestive enzymes, the gut microbiota in the stomach and colon differ from one another. Factors such as antibiotic use, sickness, stress, aging, poor dietary habits, and lifestyle can all have an impact on its composition [8]. The human microbiome composition in different bodily regions is shown in Figure 1. Furthermore, we provide a comprehensive analysis of current studies that offer a unified perspective on the human microbiota in relation to human health and disease.

## Review

General dietary impacts on gut microbiota

The scientific community has discussed the concept of diet affecting the gut flora since the 1960s. More recent research has concentrated on examining the relationship between nutrition and the makeup and functionality of the gut microbiome by utilizing animal models as well as the investigation of intestinal microbiota. One method of preventing illness is through diet. For example, low-glycemic index food for diabetes, salt restriction for heart failure, and calorie restriction for obesity are examples of dietary interventions, which are common secondary and tertiary preventive methods for several diseases. Consumption patterns have a major impact on these microbial communities' makeup and functionality [9]. The gut microbiota may mediate indirect health consequences of diet. The gut microbiota composition and diversity are significantly influenced by diet. Here, the article briefly discusses the effect of general diet food on gut microbes. Dietary intake of carbohydrates may be a factor in the significant variance of gut microbial composition seen. There appears to be a favorable correlation between bacterial richness and a high-fiber diet. Therefore, dietary adjustments may be used to permanently alter the composition of the gut microbiota [10].

**Figure 2:**
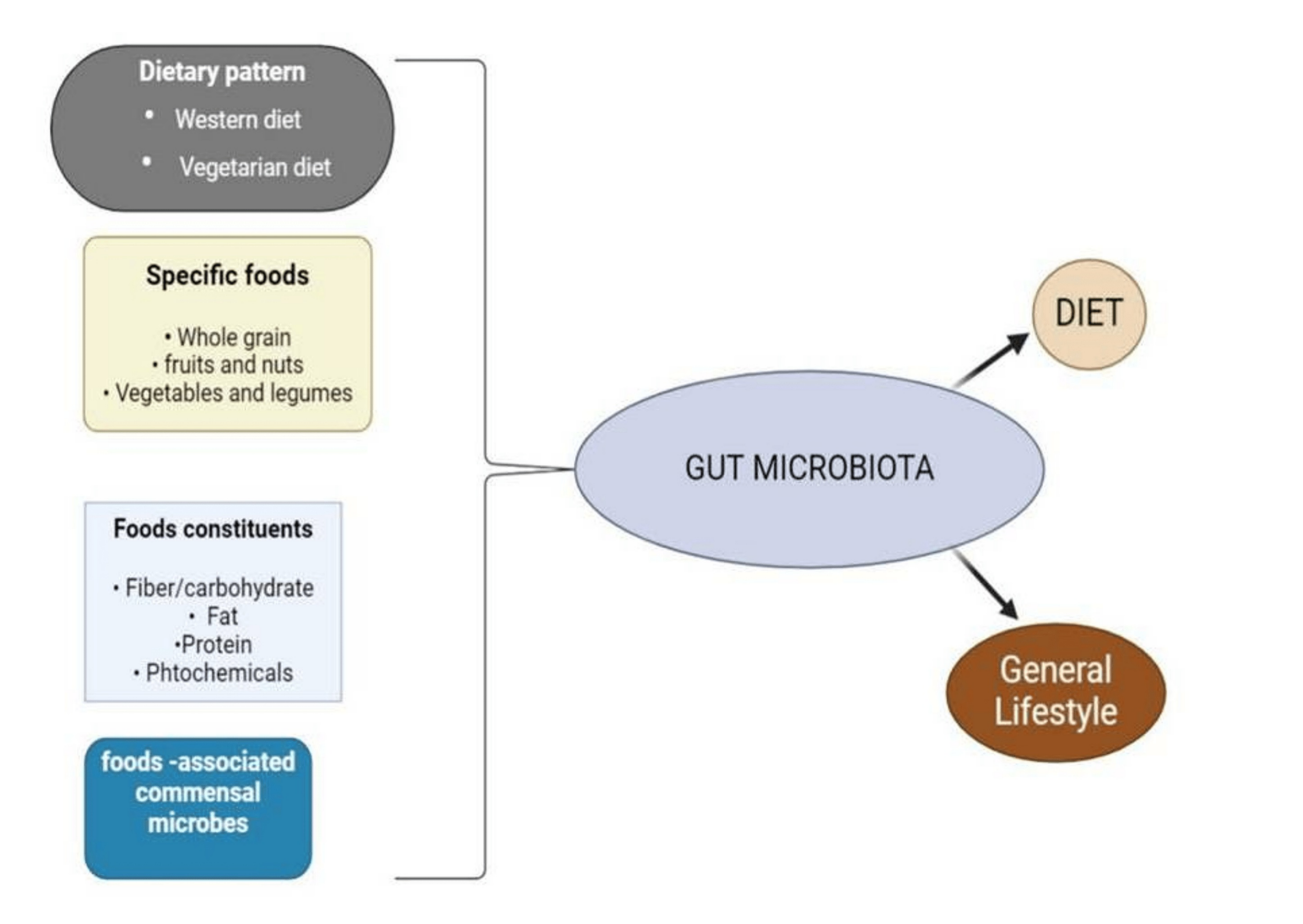
Variables that affect the human gut microbiota. Image created using BioRender (https://biorender.com/).

A diet high in protein can change the gut microbiota by reducing the amounts of *Bifidobacterium* and *Lactobacillus*, the two commonly thought to be good bacteria, while increasing the number of microorganisms that ferment proteins, such as *Bacteroides* and *Clostridium* species. Plant proteins have a greater positive effect on the diversity of gut microbiota, whereas a diet high in animal proteins typically increases the abundance of *Bacteroides* and decrease *Prevotella*. Polyphenols, which are present in fruits, vegetables, tea, coffee, and wine, are broken down by gut bacteria into bioactive substances that can alter the makeup and activity of microorganisms to improve the health of the host, as shown in Figure 2 [11]. A diet characterized by more intake of red meat, animal fat, high sugar, and low-fiber food has been linked to a higher population of *Bacteroides* phylum, primarily those that break down mucin, as well as *Ruminococcus*. Large dietary changes can be quickly absorbed by the gut microbiota, and a brief dietary change influences the species composition but not the distribution of enterotypes. Both short-term and long-term dietary modifications have been linked to particular alterations in the gut microbiota [12].

Effects of junk food on gut microbiota

Junk food includes processed food, additives, preservatives, artificial flavors, and excessive levels of sugar and saturated fats. It is low in fiber, disrupts digestion, and is deficient in vital nutrients, which results in weight gain and altered metabolism. How different types of junk food can adversely affect the gut microbiota is described in Table 1.

**Table 1:** Different types of junk food that can adversely affect the gut microbiota composition and contribute to various health issues. Table created by the authors and adapted from Turnbaugh et al. (2009) [13]. SCFA: short-chain fatty acid

Type of junk food	Effect on gut microbiota	Description	Food examples	Health consequences
High-fat diet	Decreased diversity	Reduction in overall microbial diversity	Fried chicken, burgers, pizza	Inflammation, metabolic syndromes, cardiovascular diseases
	Increased *Firmicutes*	Higher *Firmicutes* to *Bacteroidetes* ratio	-	-
	Reduction in beneficial bacteria	Decrease in beneficial beneficial bacteria such as *Bifidobacteria* and *Lactobacilli*	-	-
High-sugar diet	Increased pathogens	Overgrowth of pathogenic bacteria like *Clostridium difficile*	Candy, soda, pastries, sweetened cereals	Obesity, diabetes, gut inflammation
	Reduced SCFAs	Decrease in beneficial SCFAs due to lower fiber fermentation	-	-
	Microbiota imbalance	Dysbiosis characterized by a reduction in beneficial bacteria	-	-
Processed food	Additives impact	Emulsifiers and preservatives can disrupt microbiota	Instant noodles, packaged snacks, processed meat, frozen meals	Gut inflammation, metabolic disorders, digestive issues
	Artificial sweeteners	Can alter gut bacteria composition and lead to glucose intolerance	Diet soda, sugar-free candy, low-calorie processed food	-
	Reduced fiber	Low-fiber content reduces beneficial SCFA production	-	-

Effects of sleep disturbances on gut microbiota

The correlation between GI dysfunction and irregular sleep patterns in previously published statistics suggests that gut microbiota's impact on brain function may be involved. Half of the genes in our body that alternate between turning on and off regularly are also governed by circadian rhythms, which are widely recognized to include the sleep-wake cycle. Diet, stress, GI issues, mental health conditions, and other things can all interfere with it. More focus has been placed on poor sleep, which affects the gut microbiota, as shown by the critical role played by the brain-gut-microbiota axis in sleep intensification. The effect of sleep on gut microbiota has been the subject of more recent research, which has concentrated on common aberrant sleep modes like insomnia, obstructive sleep apnea, sleep deprivation and fragmentation, and circadian rhythm sleep-wake disorders [14].

Current research indicates that shift work, sleep deprivation, and circadian clock misalignment alter the structure of microbial communities and the expression of genes related to the clock. The composition and variety of the gut microbes in mice can also be altered by disrupting their sleep cycles. These results imply that circadian genes may have an impact on the microbiota in the gut. Disorders related to circadian rhythms seem to disrupt the balance of the gut microbiota, and this kind of disruption is linked wih the development of metabolic syndromes. Although there is variation in the results of different investigations, particularly when it comes to the precise modifications in the diversity and composition of gut microbiota, it is evident that sleep disturbance has negative effects on gut microbiota, including disturbance of gut homeostasis and a decline in gut microbiota variety. Epithelial barrier permeability and changes in gut microbes can also result from sleep deprivation. Human dysbiosis is linked to sleep loss, and microbiota configuration may be a factor in the development of metabolic abnormalities [15].

Variations in permeability of the gut, systemic immune activation, inflammation, energy harvesting, and bacterial diversity all impact sleep patterns. In the mouse light-dark cycle, the quantity of gut microbiota and the abundance of unique species like *Bacteroidetes* and *Clostridia* fluctuate. The highest bacterial load is seen during the active phase when the phylum *Firmicutes* is highly abundant, and the lowest bacterial load is found during the rest phase. The microbial community structure and gene expression of the circadian clock are altered by sleep deprivation, shift work, and circadian clock misalignment. The control of the expression of genes in the human organism by disruption of epigenetic gene modulation (i.e., through methylation) is another significant unique gut-brain axis mechanism. Although circadian signals influence the gut microbiota, the gut microbiota also has an impact on the expression of clock genes. Serum metabolite oscillations are a consequence of the gut microbiota's circadian rhythms, which are linked to changes in transcription and epigenetics in peripheral tissue. Studies on sleep deprivation in humans and animals have shown that sleep disorders are linked to changes in the expression of the human clock gene, which has a critical impact on the neurobiological reactions to stress [16].

Lack of sleep, shift work, and disruptions to circadian rhythms have all been associated with alterations in the structure of microbial communities. At the taxonomic level, *Corynebacterium,* which is present in the gut, has been noted for its ability to produce serotonin, while certain genera like *Sutterella* and *Neisseria* show positive correlations, and others such as *Blautia* and *Parasutterella* show negative correlations with sleep quality. This suggests that *Corynebacterium* may play a key role in promoting sleep as serotonin is known to regulate sleep. Modifying the sleep habits alters the composition and variety of the gut microbiota. These results imply that the gut microbiota may be impacted by circadian genes. The structure and function of the gut microbial community show regular variations. As a result, during the day, the epithelium of the gut is exposed to various species of bacteria and their metabolites. Consequently, the microbiota's circadian rhythms influence metabolite level oscillations, epigenetic changes, and the expression of hosts circadian clock genes [15].

Interplay between diet and sleep and effects on gut microbiota

When adopting fermented food-based therapies aimed at modulating the microbiota-gut-brain axis, it is crucial to take these considerations into account. A person's diet and lifestyle choices have an impact on their gut flora, immune system, and brain throughout their lives. Therefore, one subset of dietary intervention options that can be used to increase this bidirectional communication is fermented food. The metabolites produced by the gut microbial population have the ability to alter host health as well as the quality of the gut and blood-brain barrier, which in turn affects the amount of pro-inflammatory stimuli that enter the systemic circulation. Additionally, since antibiotic medication attenuates this impact, dietary polyphenols can mitigate the cognitive deficits associated with sleep deprivation via the microbiota gut-brain axis. Consuming polyphenols really guards against these cognitive deficits by means of metabolites that are dependent on microbes. This illustrates how changing the amounts of bacterial metabolites in food may affect sleep via the gut-brain-microbiota axis [14].

Numerous psychiatric, inflammatory, and metabolic diseases coexist with insomnia. It shows a drop in SCFA-producing bacteria. Insomniacs also exhibit a decline in the diversity and richness of their gut microbiomes. Moreover, these people exhibit elevated levels of the cytokine interleukin-1β that causes inflammation. With regards to sleep and probiotics, new research indicates that taking *Lactobacillus* supplements can enhance sleep quality and lessen the negative consequences of sleep deprivation. These supplements also raise the levels of several microbe metabolites, which enhance sleep quality and guard against stress-related sleep disturbances [17].

Mitigation strategies for gut microbiota

Strategies for gut microbiota focus on restoring and maintaining a healthy microbial balance to prevent and treat health issues related to dysbiosis (microbial imbalance). The microbiota can be influenced by diet in two ways: (a) by incorporating live microorganisms (probiotics) that are resistant to digestion (for example, bile acids, digestive enzymes, and stomach acid) and enter the colon where they (temporarily) implant, develop, and start metabolism; and (b) by incorporating non-digestible substrates (prebiotics) that are resistant to metabolism and enrich the colon by boosting the local bacteria's development and metabolism.

Prebiotics

Prebiotics are defined as "selectively fermented ingredients that allow specific changes, both in the composition and/or activity in the GI microflora that confer benefits upon host well-being and health." Their capacity to promote *Bifidobacteria* selectively is a frequently checked characteristic of prebiotics. Fiber for diet can be found in vegetables, fruits, grains, and nuts. Even though fiber in general is thought to be good for GI health, certain kinds of food fiber like inulin, fructo-oligosaccharides, and galacto-oligosaccharides are also considered to be prebiotics; prebiotics are defined as “a substrate that is selectively used by host microorganisms conferring a health benefit” [18].

Growth of lactic acid bacteria, *Lactobacillus*, and *Bifidobacterium* can be caused by prebiotic dietary intake. Prebiotics are long or short-chain carbohydrates that are fermented into SCFAs and gas in the colon rather than being absorbed in the small bowel [19].

Probiotics

"A live microbial food ingredient that is beneficial to health" is the definition of a probiotic. Probiotics from the genera *Lactobacilli* and *Bifidobacteria* are likely the most researched. These genera have a long history of safety, having been employed for many years in the fermented food business and, more recently, in probiotic food. The effect of probiotic therapy in the treatment of GI illnesses and disorders has been studied. The gut microbiome needs to be kept in balance because it is important for the development of several diseases. The GI epithelium serves as the initial site of contact between the host and pathogenic bacteria. Any infection or invasion of this mucosa will trigger an immune response. A number of GI conditions, including colon cancer, irritable bowel syndrome, and inflammatory bowel disease, as well as parenteral conditions such as allergens, asthma of the bronchi, and cystic fibrosis (CF), are associated with changes in the diversity and makeup of microflora [16].

Pseudomembranous colitis is known to be caused by *Clostridioides difficile* and is frequently brought on by the use of antibiotics, particularly broad-spectrum medications. Using traditional methods to treat the illness can be challenging. Probiotics made from yeast have produced positive outcomes. *Saciharornycer houlardii *was used to treat a large number of patients in two well-controlled trials. The number of patients getting antibiotic-associated diarrhea (AAD) was significantly reduced as a result of this treatment. Although the exact method of action is yet unknown, it has been hypothesized that the yeast may be reducing the effects of *Clostridium difficile* by destroying gut wall receptors involved in toxin or cell adhesion. Probiotics have the potential to modify the microbiota in the gut of humans by encouraging the development of lactic acid-producing bacteria and inhibiting the growth of harmful bacteria such as *Desulfovibrio*. A specific study found that on pre-treatment with the probiotic bacteria Bifico (*Bifidobacterium infantis*), the number of *Lactobacilli* and colitogenic bacteria, such as *Desulfovibrio*, *Mucispirillum*, and *Odoribacter*, were reduced by *Lactobacillus acidophilus*, *Enterococcus faecalis*, and *Bacillus cereus*. As a result, the chance of colitis-related tumor growth and cancer decreased [9].

Probiotic yeast *Saccharomyces boulardii* was found to significantly increase *Bacteroidetes* abundance and decrease *Firmicutes*, *Proteobacteria*, and *Tenericutes* abundance in another animal study. These findings were correlated with changes in host metabolism and suggested a potential role for *Saccharomyces boulardii* in the treatment of obesity and type 2 diabetes. These results suggest that probiotics may control and replenish the balance of gut microbiota through limiting the spread and activity of dangerous bacteria while encouraging the creation and activity of beneficial bacteria. Probiotics may slow the growth of infections by competing with them for colonization sites and producing toxins and SCFAs, but the underlying mechanism of their effect on gut microbes modification is unknown [20].

## Conclusions

The complex interplay of food consumption, sleep disorders, and gut microbiota highlights the significance of lifestyle choices in preserving gut health. The gut's diversity of microbes and metabolic processes are greatly influenced by dietary decisions, and sleep disturbances can intensify these effects by inducing stress responses and dysregulating the circadian rhythm. Knowing these dynamics makes it easier to see how focused treatments like food changes and good sleeping habits can support a healthy gut microbiota and advance general health and well-being. Further research into these relationships should create new opportunities for customized health strategies that optimize the composition and functionality of the microbiota in the gut.
